# 1706. Risk factors for infections caused by extended spectrum beta lactamase (ESBL) producing and Carbapenem-resistant Enterobacterales (CRE) in pediatric critical care settings: A case control study

**DOI:** 10.1093/ofid/ofad500.1539

**Published:** 2023-11-27

**Authors:** Amr Omar, Basim Asmar, Nahed M Abdel-Haq

**Affiliations:** Children's Hospital of Michigan, Detroit, Michigan; Children's Hospital of Michigan. Central Michigan University, Detroit, Michigan; Children's Hospital of Michigan. Central Michigan University, Detroit, Michigan

## Abstract

**Background:**

Infections due to Gram negative resistant bacterial pathogens are a major concern in intensive care units. The purpose of this study is to determine the risk factors for developing ESBL-producing and CRE infections in pediatric patients in critical care settings (PICU and NICU).

**Methods:**

A retrospective case control study of infections due to ESBL-producing and CRE during a 5-year period (2016-2021) at Children’s Hospital of Michigan, Detroit. All patients had positive cultures during their PICU/NICU stay. Control cases were matched for age, infection site and year of infection but their cultures grew Enterobacterales that were non-ESBL or carbapenemase producers.

**Results:**

A total of 58 patients with ESBL/Carbapenemase-producing Enterobacterales (cases) and 59 control patients were included. Majority of organisms were *E. coli* and *Klebsiella* species recovered from respiratory tract. There was no difference in both groups in regards to age, gender, race or length of hospital stay (p >0.3). There was no difference in prior hospital admission, PICU or NICU stay during the previous 6 months, indwelling devices, comorbid conditions, or surgery in the last 6 months (p >0.3). However, the presence of gastrostomy tube was more frequent in the cases group (29.3%) than the control group (15.3%) (p=0.078). Risk factors by univariate analysis included history of antibiotic use in last 6 months (93% in cases vs 73% in controls; p=0.006), cephalosporins use > 14 days (67% vs 31.2%; p=0.006),Trimethoprim/Sulfamethoxazole treatment duration > 14 days (11.1% vs 0%; p=0.012), carbapenem treatment duration > 14 days (14% vs 1.9%; p=0.027), and vancomycin use > 14 days (43.6% vs 21.6%; p=0.053). However, multivariate logistic regression analysis showed that antibiotic usage in the last 6 months was the single best predictor for cases with odds ratio of 5.02-fold (95% CI 1.56-16.13) (*p*=0.007).

**Conclusion:**

Prior broad spectrum antibiotic usage was significantly associated with acquisition of ESBL and carbapenemase producing Enterobacterales. The study further underscores the need for optimizing antimicrobial stewardship practices to limit unnecessary and prolonged use of antibiotics to prevent the emergence of resistant pathogens.

**Disclosures:**

**All Authors**: No reported disclosures

